# Adrenal Schwannoma: Case Description and Diagnostic Pointers of a Rare Disease

**DOI:** 10.7759/cureus.34485

**Published:** 2023-02-01

**Authors:** Shiraz A Mohd Ziauddin, Aditya P Sharma, Sudheer K Devana, Kim Vaiphei

**Affiliations:** 1 Urology, Postgraduate Institute of Medical Education & Research, Chandigarh, IND; 2 Histopathology, Postgraduate Institute of Medical Education & Research, Chandigarh, IND

**Keywords:** s-100, benign nerve sheath tumors, histopathology and immunohistochemistry, adrenal schwannoma, unilateral adrenalectomy

## Abstract

Benign nerve sheath tumours such as schwannomas commonly involve the peripheral and cranial nerves. A schwannoma in the adrenal gland is a very rare occurrence, which arises from the adrenal medulla. Its most common presentation is a non-functional incidentaloma. It does not have any unique imaging characteristic distinguishing it from other adrenal masses; hence, its diagnosis is usually confirmed by final histopathology. In this report, we present two cases of an adrenal schwannoma for which we anticipated an unusual diagnosis, which was confirmed through adrenalectomy on histopathology.

## Introduction

Nerve sheath tumours such as schwannomas can arise from cranial and peripheral nerves of the head, neck, and extremities, retroperitoneum, or adrenal medulla [1,2]. They are mostly benign; however, although very rarely, malignant tumours have been reported. An adrenal schwannoma is an extremely rare pathology that is detected as an incidentaloma and constitutes less than 0.2% of the 0.5-5% of all retroperitoneal schwannomas [3].

Clinically, most retroperitoneal schwannomas present with dull flank pain and abdominal discomfort or are detected incidentally during imaging for unrelated symptoms or at the time of autopsy [4,5]. The histological hallmark of the Schwann cell origin of the tumour is S-100 protein positivity detected through immunohistochemical analysis [6]. In this report, we present two cases of adrenal tumours where the tumours were detected during the evaluation of left flank pain and the diagnosis of an adrenal schwannoma was later established on final histopathology. Pointers for the diagnosis of this rare entity are discussed, and a brief review of the literature related to adrenal schwannoma has also been presented.

## Case presentation

Case 1

**Figure 1:**
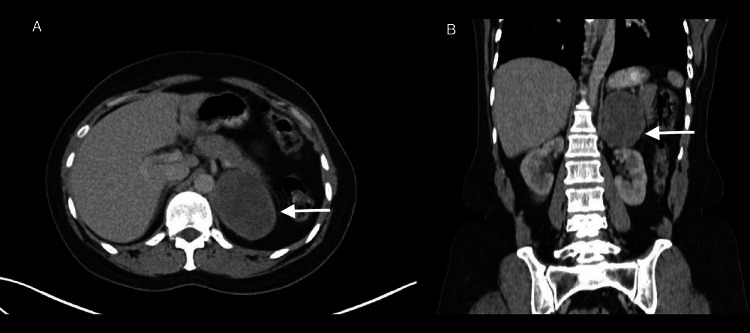
Contrast enhanced-computerized tomography (CECT) abdomen images A. axial image showing a 6.5 ×5 × 6.5 cm left suprarenal mass with peripheral enhancement and B. Coronal section showing left kidney inferior displacement by the mass effect of the tumour (white arrow).

A 44-year-old female with no comorbidities presented to our hospital with occasional left flank pain for three months. Initial ultrasound evaluation showed a left suprarenal mass of 6.5 × 6 cm with mixed echogenicity. Contrast enhanced-computerized tomography (CECT) of the abdomen was suggestive of a well-defined soft tissue lesion of size 6.5 × 5 × 6.5 cm in the left suprarenal region, with peripheral enhancement without any fat attenuation or calcification (Figure 1).

 

**Figure 2:**
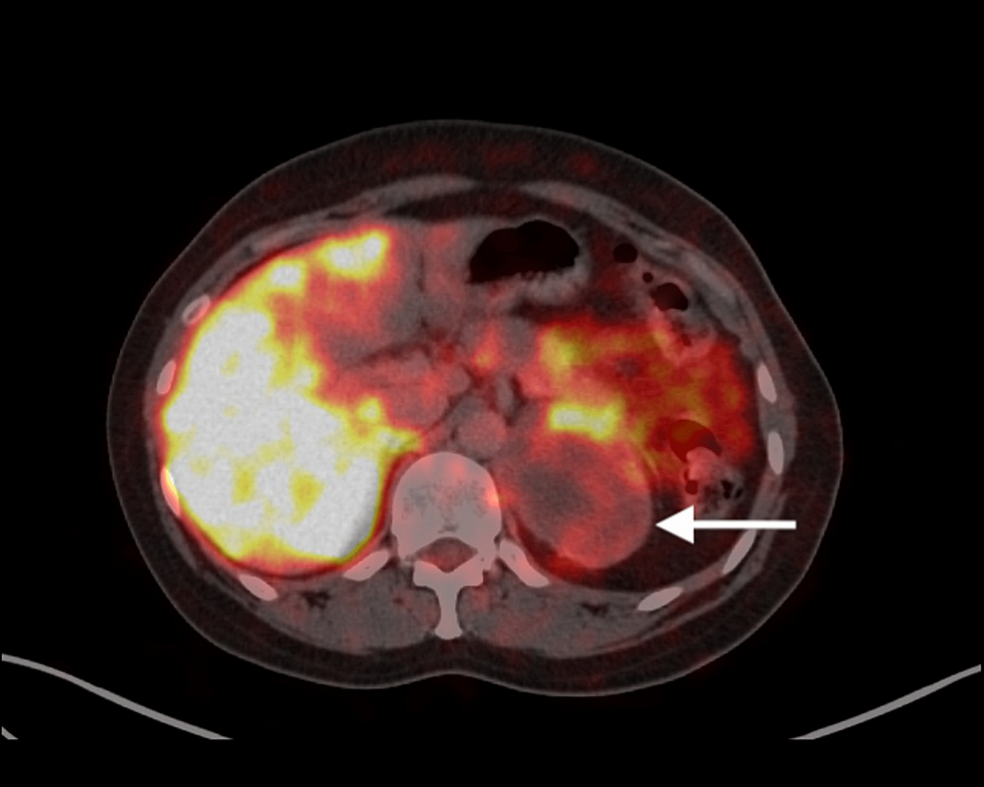
Axial section of fluorodeoxyglucose positron emission tomography (FDG PETCT) showing faint tracer uptake of the left suprarenal mass (white arrow).

Fluorodeoxyglucose positron emission tomography (FDG PETCT) showed patchy tracer avidity in the left suprarenal region {Standard uptake value (SUV) max-13.5}, along with predominant necrotic areas with no evidence of any metastatic lesions elsewhere in the body (Figure 2).

**Figure 3:**
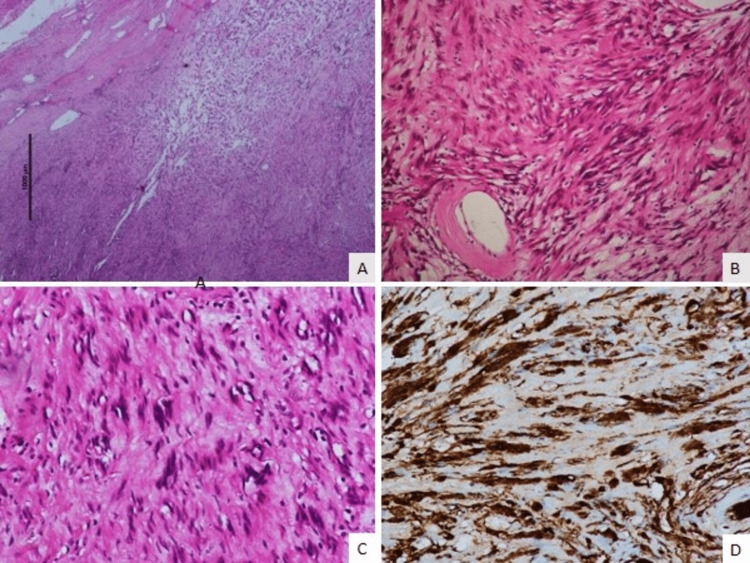
Panel of histological photomicrographs showing A – low-power photomicrograph of the tumour showing the capsule at the upper right-hand corner of the picture and the tumour cells exhibiting paler hypocellular and darker hypercellular areas (HEx50) {Hematoxylin and Eosin}; B – The spindle-shaped tumour cells are arranged in fascicles with no nuclear pleomorphism (HEx150); C – Higher-power photomicrograph of the tumour showing the nuclei arrange din palisaded pattern (HEx250); D – Medium-power photomicrograph showing cytoplasmic positivity for S-100 in immunohistochemistry (peroxidase anti-peroxidase x250).

Routine blood and hormonal workups, including serum cortisol, dehydroepiandrosterone sulfate (DHEAS), metanephrine, and normetanephrine, along with 24-hour urinary metanephrine and normetanephrine, were within normal limits, suggesting non-functional adrenal tumour. Laparoscopic adrenalectomy was performed, and intraoperatively, there was a well-encapsulated 6 × 6 cm suprarenal mass with multiple feeding vessels mainly from the medial side. Dissection was difficult, and intraoperatively, the tumour mass was felt to be hard and gritty when lifted using the suction device or grasper. The gross excised specimen on the cut section showed a homogenous solid component with yellowish nodules in between. Again, a gritty sensation was felt during cutting the specimen. The intraoperative and immediate post-operative phase was uneventful, and the patient was discharged on postoperative day three. The final histopathology revealed a well-encapsulated tumour composed of spindle cells with infrequent mitosis, areas of hyalinization, and cystic degeneration. Immunohistochemistry showed diffuse S-100 nuclear positivity pointing to a diagnosis of adrenal schwannoma (Figure 3).

. After two months of follow up the patient is symptomatically relieved with no evidence of any tumour recurrence or metastasis.

 

Case 2

**Figure 4:**
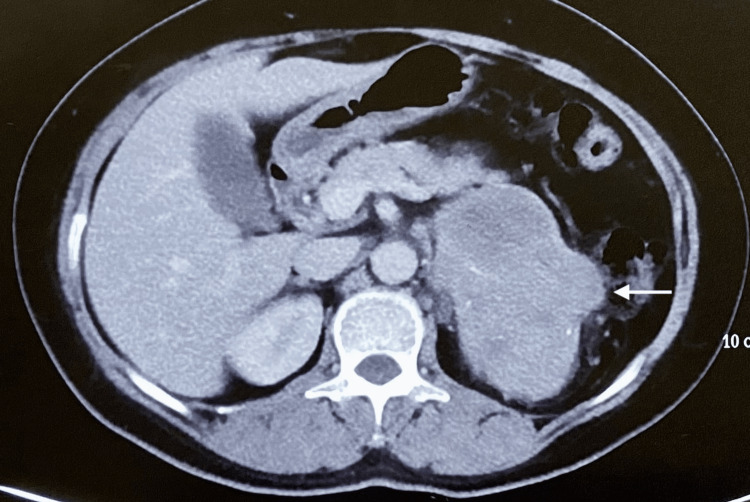
Axial section showing large heterogeneously enhancing mass lesion of approximately 10.6 × 9.6 × 7.5 cm in size in the left suprarenal region (white arrow).

A 45-year-old hypertensive female with a recent history of left flank pain on evaluation was found to have a large (11 × 10 × 7 cm) left suprarenal mass on the CECT abdomen (Figure 4).

**Figure 5:**
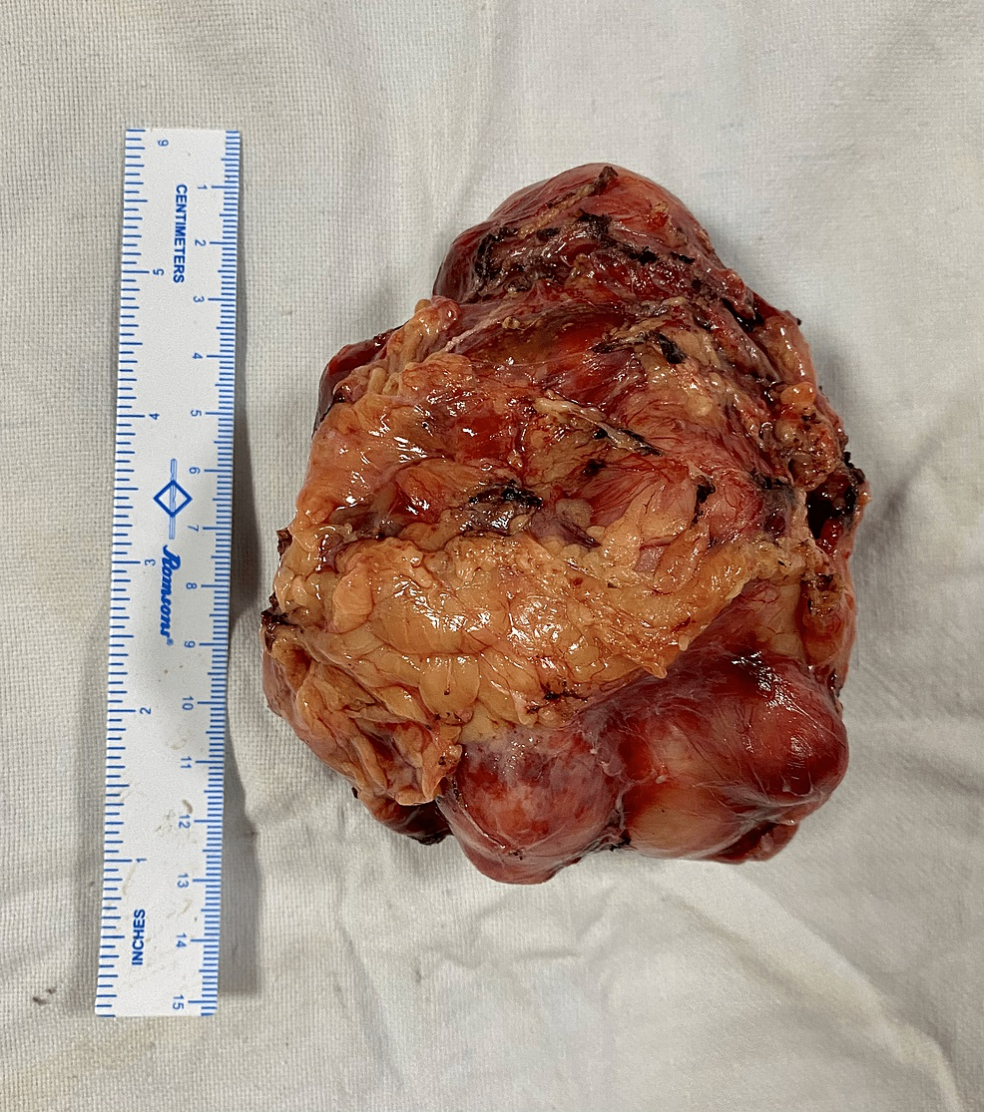
Gross specimen of hard left suprarenal mass of 12x7 cm excised.

The CT findings were confirmed through an FDG-PET CT scan that showed an FDG avid mass of standardized uptake value (SUV) max-11.4 along with FDG-avid paraaortic and hilar lymph nodes. Blood levels of aldosterone, cortisol, DHEAS and urinary 24-hour metanephrine, and normetanephrine levels were within normal limits. After medical blood pressure optimization, the patient was taken up for left-open adrenalectomy and hilar lymphadenectomy. Intraoperatively, a stony hard left suprarenal mass of size 12 × 7 cm was found with lateral and inferior displacement of the ipsilateral kidney and maintained fat planes with adjacent organs. The suprarenal mass along with enlarged hilar lymph nodes was excised, and the specimen was sent for histopathological analysis (Figure 5).

The post-operative period was uneventful. The histopathology again revealed adrenal schwannoma with lymph nodes negative for metastasis.

## Discussion

Schwannomas are benign nerve sheath tumours arising from cranial and peripheral nerves, with the VIII cranial nerve being the most common site. Most frequently found in the head and neck region, followed by the upper and lower extremities, trunk, gastrointestinal tract, liver, pancreas, kidney, brain, heart, and retroperitoneum [7]. Schwannomas constitute 1-5% of all retroperitoneal tumours [8,9]. In a report series on 1111 adrenal incidentalomas from Poland, only two cases were determined as adrenal schwannomas, which is 0.18% of the total cases [3], underscoring the rarity of the lesion. Their aetiology still remains an enigma [10]. While there are no age-based differences in incidence, the tumour predominantly occurs in females during the second to fifth decade of their lives. Both of our patients were females who were in the fifth decade of their lives.

Retroperitoneal schwannomas are most commonly discovered incidentally in patients being evaluated for vague and non-specific abdominal pain. They are usually >4cm on diagnosis, as seen in our cases as well. Imaging through CT and MRI of a retroperitoneal mass localized to the adrenal region had differential diagnoses of adrenal adenoma, pheochromocytoma, myelolipoma, adrenocortical carcinoma, ganglioneuroma, and adrenal metastasis. Recently, based on the imaging characteristics through multi-detector computed tomography (MDCT), a well-defined unilateral mass with cystic degeneration septa and a characteristic progressive, contrast-enhanced pattern has been proposed as a unique hallmark of adrenal schwannoma, as seen in one of our cases [11].

Functional evaluation through hormonal workup is always recommended for the management of any adrenal mass, including adrenal schwannoma. However, invariably the functional evaluation is negative in schwannomas. Both of our cases were suspicious of malignant adrenal neoplasm owing to the large size and imaging characteristics. PET CT is highly sensitive and specific for detecting adrenal malignancy along with metastases. We routinely obtained PET scans for all adrenal tumours >4 cm for which CT is not categorically suggestive of myelolipoma. PET CT helps rule out distant metastases. In both of our cases, PET scans showed high SUV max (>4) in the region of suprarenal mass suggestive of mitotic pathology.

Surgical excision remains the gold standard treatment and diagnosis is established only by histopathology [10,12]. Surgical resection is the only definitive method to establish the diagnosis along with relieving patient symptoms. Laparoscopic adrenalectomy, either transperitoneal or retroperitoneal, is currently the most frequent treatment modality in the era of minimally invasive surgery whenever surgical expertise is present. The open approach has the advantage of allowing for a thorough abdominal inspection for any metastatic deposits and lymph nodal dissection as was done in our second case where the tumour was large and regional lymphadenopathy pointed to an adrenocortical carcinoma [13,14]. However, in the first case, we describe a seemingly benign 6.5 × 5 × 6.5 cm left adrenal tumour, the patient was operated on laparoscopically, followed by a smooth postoperative phase and an early discharge with a quick return to the routine lifestyle. The tumours in both cases were hard and gritty during cutting and showed whirling, which was suggestive of a schwannoma. This is a key feature that differentiates schwannomas from other benign adrenal pathologies.

A definitive diagnosis of schwannoma is obtained through the final histopathology with the tumour showing spindle cells having infrequent mitoses, along with areas of hyalinization and cystic degeneration with immunohistochemical S-100 antigen, vimentin, collagen IV, laminin positivity and keratin, desmin, CD 34 & CD117 negativity. Diffuse positivity for S-100 protein in the tumour cell cytoplasm is characteristic of tumours of neuroectodermal origin, along with unique nuclear freeze zones called the “Verocay bodies” [6].

Malignant schwannomas are infrequent but are typically associated with Von Recklinghausen’s disease or Neurofibromatosis 1 (NF1) in 5-18% of the cases [15], with concurrent loss of heterozygosity of NF-1 and p53 mutation [16].

## Conclusions

An adrenal schwannoma is a rare entity detected only after surgical excision of an adrenal incidentaloma and its histopathological analysis. Laparoscopic excision and open excision of the tumour are the most favourable procedures, with one being chosen based on feasibility. Non-functional nature of the tumour and the hard, gritty sensation during tumour handling and cutting of the specimen may be suggestive of an adrenal schwannoma; however, the definitive diagnosis is achieved through the final histopathological examination of the excised specimen.

## References

[1] Ohta I, Lin PH, Rau CL, Wang KC (2007). Evaluation of perinephric, retroperitoneal schwannomas: case report and review of the literature. South Med J.

[2] Brady KA, McCarron JP Jr, Vaughan ED Jr, Javidian P (1993). Benign schwannoma of the retroperitoneal space: case report. J Urol.

[3] Kasperlik-Zaluska AA, Roslonowska E, Slowinska-Srzednicka J (2006). 1,111 patients with adrenal incidentalomas observed at a single endocrinological center: incidence of chromaffin tumors. Ann N Y Acad Sci.

[4] Cury J, Coelho RF, Srougi M (2007). Retroperitoneal schwannoma: case series and literature review. Clinics (Sao Paulo).

[5] Yang CY, Chou CW, Lin MB, Li CF (2009). Schwannomas of the left adrenal gland and posterior mediastinum. J Chin Med Assoc.

[6] Jakowski JD, Wakely PE Jr, Jimenez RE (2008). An uncommon type of adrenal incidentaloma: a case report of a schwannoma of the adrenal medulla with cytological, histological, and ultrastructural correlation. Ann Diagn Pathol.

[7] Sharma SK, Koleski FC, Husain AN, Albala DM, Turk TM (2002). Retroperitoneal schwannoma mimicking an adrenal lesion. World J Urol.

[8] Garg S, Mathew M, Goel T (2007). Adrenal schwannoma: a case report and review of literature. Indian J Pathol Microbiol.

[9] Korets R, Berkenblit R, Ghavamian R (2007). Incidentally discovered adrenal schwannoma. JSLS.

[10] Ji JH, Park JS, Kang CM, Yoon DS, Lee WJ (2017). Laparoscopic resection of retroperitoneal benign neurilemmoma. Ann Surg Treat Res.

[11] Zhang YM, Lei PF, Chen MN, Lv XF, Ling YH, Cai PQ, Gao JM (2016). CT findings of adrenal schwannoma. Clin Radiol.

[12] Zeiger MA, Thompson GB, Duh QY (2009). The American Association of Clinical Endocrinologists and American Association of Endocrine Surgeons medical guidelines for the management of adrenal incidentalomas. Endocr Pract.

[13] Pinto D, Kaidar-Person O, Cho M, Zundel N, Szomstein S, Rosenthal RJ (2008). Laparoscopic resection of a retroperitoneal degenerative schwannoma: a case report and review of the literature. Surg Laparosc Endosc Percutan Tech.

[14] Descazeaud A, Coggia M, Bourriez A, Goëau-Brissonnière O (2003). Laparoscopic resection of a retroperitoneal schwannoma. Surg Endosc.

[15] Rattier B, Desrousseaux B, Dereux HJ, Atat I, Ampe J (1990). [Benign retroperitoneal pelvic schwannoma. Apropos of 2 cases]. J Chir (Paris).

[16] Baisakh MR, Mohapatra N, Adhikary SD, Routray D (2014). Malignant peripheral nerve sheath tumor of adrenal gland with heterologus osseous differentiation in a case of von Recklinghausen's disease. Indian J Pathol Microbiol.

